# Growth, Health, and Gut Microbiota of Female Pacific White Shrimp, *Litopenaeus vannamei* Broodstock Fed Different Phospholipid Sources

**DOI:** 10.3390/antiox11061143

**Published:** 2022-06-10

**Authors:** Xiaolong Liang, Xiaolong Luo, Hongxing Lin, Fenglu Han, Jian G. Qin, Liqiao Chen, Chang Xu, Erchao Li

**Affiliations:** 1 Key Laboratory of Tropical Hydrobiology and Biotechnology of Hainan Province, Hainan Aquaculture Breeding Engineering Research Center, College of Marine Sciences, Hainan University, Haikou 570228, China; 19095134210017@hainanu.edu.cn (X.L.); luoxiaolong0618@163.com (X.L.); linhongxing688@163.com (H.L.); hanfenglu@163.com (F.H.); 2 School of Biological Sciences, Flinders University, Adelaide, SA 5001, Australia; jian.qin@flinders.edu.au; 3 School of Life Sciences, East China Normal University, Shanghai 200241, China; lqchen@bio.ecnu.edu.cn

**Keywords:** *Litopenaeus vannamei*, broodstock, phospholipid, antioxidant, gut microbiota

## Abstract

Phospholipids have an important antioxidant effect on animals. The effects of different dietary phospholipid sources on the growth, antioxidant activity, immunity, and gut microbiota of female broodstock of Pacific white shrimp *Litopenaeus vannamei* were investigated. Four isoproteic and isolipid semi-purified diets containing 4% soybean lecithin (SL), egg yolk lecithin (EL), or krill oil (KO) and a control diet without phospholipid supplementation were fed to female broodstock of *L. vannamei* (34.7 ± 4.2 g) for 28 days. The growth performance, antioxidative capacity, and innate immunity of the female broodstock fed phospholipid supplemented diets were improved regardless of sources compared with the control shrimp. The effects on growth and antioxidant capacity in female shrimp fed the KO diet were highest. The innate immunity of female shrimp fed the EL and KO diets were significantly higher than shrimp fed the SL diet. Dietary phospholipid supplementation increased gut microbiota diversity and richness, and the Chao1 and ACE values in the KO group were significantly higher than in the control group. The richness of Proteobacteria, *Photobacterium*, and *Vibrio* decreased, whereas the richness of Firmicutes and Bacteroidetes increased in the shrimp fed the KO diet compared with the shrimp fed the SL and EL diets. The interactions of gut microbiota in shrimp fed the KO diet were the most complex, and the positive interaction was the largest among all the treatments. The functional genes of gut microbiota in shrimp fed the KO diet were significantly enriched in lipid metabolism and terpenoid/polyketide metabolism pathways. Spearman correlation analysis showed that *Fusibacter* had significantly positive correlations with antioxidant activity (total antioxidant capacity, superoxide dismutase, glutathione peroxidase), immune enzyme activity (phenoloxidase and lysozyme), and immune gene expression (C-type lectin 3, Caspase-1). All findings suggest that dietary phospholipids supplementation can improve the growth and health status of female *L. vananmei* broodstock. Krill oil is more beneficial in improving the antioxidant capacity and innate immunity than other dietary phospholipid sources. Furthermore, krill oil can help establish the intestinal immune barrier by increasing the richness of *Fusibacter* and promote the growth of female shrimp. *Fusibacter* may be involved in iron metabolism to improve the antioxidant capacity of female shrimp.

## 1. Introduction

The Pacific white shrimp, *Litopenaeus vannamei*, is the most important shrimp species cultured globally in scale and production [1]. The continuous supplementation of healthy broodstock supports large-scale, healthy culture of *L. vannamei*. However, during the practical culture of *L. vannamei* broodstock, diseases frequently occur and significantly limit the healthy and sustainable development of the industry [2,3]. Furthermore, the *L. vannamei* broodstock industry has suffered severe economic losses. In addition, the health status of shrimp broodstock can directly affect the activity and growth of shrimp larvae. Therefore, ensuring the health of *L. vannamei* broodstock is of great significance to the sustainable development of the shrimp culture industry worldwide.

After years of development, the *L. vannamei* broodstock industry has established a more efficient biosecurity system free of specific pathogens [4]. Improving reproductive performance is a fundamental goal of developing the shrimp broodstock industry. To ensure the maturation and quality of shrimp gonads, many fresh polychaetes and frozen squid are fed to *L. vannamei* broodstock in various hatcheries [5]. However, these polychaetes and squids have high price, unstable quality, and often carry pathogens [2,6,7]. *L. vannamei* broodstock is easily infected by these pathogens, and a series of health problems or mass death may occur. Therefore, it is necessary to develop nutritionally balanced diets for *L. vannamei* broodstock to ensure gonadal development and the health of *L. vannamei* broodstock.

Research on the nutrition of *L. vannamei* broodstock has been limited to dietary lipids, fatty acids, and vitamins. Previous studies have shown that dietary lipid levels can significantly affect *L. vannamei* ovary development, but the optimal dietary fatty acid level is inconsistent among previous studies on *L. vannamei* broodstock [8,9,10]. Dietary 2–3% highly unsaturated fatty acids could satisfy the normal development of *L. vannamei* ovaries [8,11]. Arachidonic acid, accounting for 4.65% of the total fatty acids in feed, can significantly improve the spawning performance and larval quality of *L. vannamei* [9]. Vitamin research has mainly been conducted with vitamin E and vitamin C. Dietary 300 mg/kg of vitamin E could significantly increase the hepatopancreas index and gonadosomatic index before eyestalk ablation and significantly shorten the days to spawning after eyestalk ablation [12]. Dietary 800 mg/kg of ascorbic acid can satisfy ovarian maturation and maintain good reproductive performance in *L. vannamei* broodstock [13]. However, dietary 1000–2000 mg/kg ascorbic acid had no significant effects on immune indexes, such as total hemolymph cell count and phenoloxidase activity in *L. vannamei* broodstock after eyestalk ablation [14]. Although some nutritional research on *L. vannamei* broodstock has been carried out, information on the effect of nutrients on the health status of *L. vannamei* is still scarce.

Polychaetes and squids are rich in lipids and highly unsaturated fatty acids and are especially high in phospholipids useful to ensure the gonadal development of aquatic animals [15]. Dietary 2% soybean lecithin can meet the needs of ovary maturation of giant freshwater prawn *Macrobrachium rosenbergii* [16]. Lecithin also can upregulate the expression of vitellogenin genes in the hepatopancreas of redclaw crayfish *Cherax quadricarinatus* [17]. The gonadal index of Chinese mitten crab *Eriocheir sinensis* fed 2.4% soybean lecithin was the highest among all the treatments [18]. In addition, as an essential nutrient for crustaceans, phospholipids play an essential role in modulating the growth and health of animals, but the dose effects of different phospholipids are different. The specific growth rate of swimming crabs *Portunus trituberculatus* fed the egg yolk lecithin-supplemented diet was significantly higher than that of *P. trituberculatus* fed the soybean lecithin diet [19]. Dietary krill oil could improve the antioxidant activity of *E. sinensis* more efficiently than dietary soybean lecithin or egg yolk lecithin [20]. However, there is no information on the regulation of phospholipids on the health status of *L. vannamei* broodstock. The supplementation of phospholipids to the feed can enhance the glutathione metabolism of female shrimp and further improve the antioxidant capacity [21].

Therefore, based on the results of previous research, this study evaluated the effects of three phospholipids (soybean lecithin, egg yolk lecithin, and krill oil) on *L. vannamei* in terms of growth, antioxidant capacity, immunity, and gut microbiota. The purpose of this study is to find a suitable phospholipid source for the culture of healthy *L. vannamei* broodstock. The results of this study contribute to diet development for *L. vannamei* broodstock.

## 2. Materials and Methods

### 2.1. Experimental Diets

According to the nutritional studies on broodstock of *L. vannamei* [9,10] and our previous research [21], four isoproteic (52.4% crude protein) and isolipidic (14.2% crude lipid) semi-purified diets involving 4% soybean lecithin (SL), egg yolk lecithin (EL), or krill oil (KO) and a control diet (Ctrl) without phospholipid supplementation were prepared (Table 1). The crude protein, crude lipid, and fatty acids were determined by Dumas combustion, Soxhlet extraction, and gas chromatograph-mass spectrometer (GC-MS), respectively. The protein was derived from fishmeal, gelatin, and casein in the feed ingredients. In addition, the oil sources in the experimental diets mainly included fish oil, cholesterol, palm oil, and three different phospholipids. The coarse materials were ground with a grinder, crushed into powder by an 80-mesh sieve, and then weighed. The processed dry ingredients were weighed and mixed thoroughly according to the proportions in the formulations of experimental diets, followed by the supplementation of oil and water, and mixed well again. Feed pellets with a diameter of 2.5 mm were extruded by a double helix plodder (CD4-1TS, Guangdong Huagongguang Mechanical and Electrical Technology Co., Ltd., Guangdong, China) and then were air-dried at room temperature. The feed pellets were sealed in plastic bags and frozen at −20 °C before use.

### 2.2. Growth Trial and Sampling

All the female *L. vannamei* broodstock were obtained from a private company in Hainan, China. Before the experiment began, the female shrimp were acclimated in a black polypropylene barrel (diameter × height = 5.8 × 1.1 m). Subsequently, 160 shrimps (34.7 ± 4.2 g, hepatosomatic index 6.23 ± 0.25%) were randomly divided into 16 barrels (diameter × height = 1.0 × 0.9 m, four barrels per treatment at 10 shrimp/barrel) and fed the control diet for 7 days to adapt to the experimental conditions. During the 28-day culture process, the daily feeding volume was about 5.5% of the body weight, and the daily feeding was carried out at 7:30, 10:00, 13:00, 15:00, 18:00, 21:00, and 23:30 for a total of 7 times. This breeding strategy is based on the summary of our previous work [21] and the suggestions of Arshadi et al. [22] to account for a total daily supply of 5% of wet weight biomass per day. Residual food and feces were removed by siphon twice a day, and the daily water exchange was about 50%. During the whole culture experiment, the water quality parameters were controlled as follows: temperature 28~29 °C, pH 7.8~8.4, salinity 30~32, dissolved oxygen 5~6 mg/L, ammonia nitrogen 0.10~0.30 mg/L, nitrite 0.03~0.10 mg/L, and 12 h light and 12 h dark.

After the 28 days of the experiment, the shrimp were fasted for 24 h for sample collection. After anesthesia for 10 min on ice, the shrimp in each experimental bucket were weighed and measured. The hemolymph of the shrimp was drawn from the cardiocoelom and abdomen (at the first swimming foot) with a 1-mL sterile syringe and stored for 24 h at 4 °C. The hepatopancreas and midgut were frozen in liquid nitrogen and transferred to a −80 °C freeze for subsequent analysis. Considering the individual differences of gut microflora, five shrimp were taken as a sample, with a total of 5 samples in each treatment. The growth performance-related parameters were calculated as follows:

Survival (%) = (Final number/Initial number) × 100;

Condition factor (%) = Final weight/(Body length)^3^ × 100;

Weight gain (%) = (Final weight − Initial weight)/Initial weight × 100;

Specific growth rate (%, day^−1^) = [In (Final weight) − In (Initial weight)]/Culture days × 100.

### 2.3. Antioxidant Capacity Related Parameter Assays

Eight hepatopancreases per treatment, from two shrimp per barrel, were homogenized in the pre-chilled 0.86% saline solution (1:10, *w*/*v*), at a frequency of 60 Hz at 4 °C for 30 s (Tissuelyser-24, Jingxin Technology, Shanghai, China), then centrifuged at 1500× *g* for 15 min in 4 °C (SIGMA 3-18K; Sigma, Laborzentrifugen GmbH, Osterode, German). The supernatant was collected for measuring total antioxidant capacity (T-AOC), malondialdehyde (MDA), glutathione peroxidase (GSH-Px), and superoxide dismutase (SOD) using diagnostic reagent kits (Nanjing Jiancheng Bioengineering Institute, Nanjing, China). Detailed steps of the parameter assays accorded with the instructions provided by the manufacturer.

### 2.4. Immunity Related Parameters Assay

Stored hemolymph samples were centrifuged at 4500× *g* at 4 °C for 10 min. The supernatant was aspirated and stored at −80 °C for later analysis. Eight serum per treatment from two shrimp per barrel were used to determine the activity of phenoloxidase (PO) and lysozyme (LZM).

Ashida’s method [23] was improved by using levodopa as the substrate to determine phenoloxidase activity. We added 10 μL serum, 300 μL potassium phosphate buffer (0.1 mol/L, pH 6.0), and 10 μL L-dopa solution (0.01 mol/L) to the enzyme plate and the absorbance at 490 nm was read every 3 min. Under the experimental conditions, an increase of 0.001 per min of OD490 was regarded as a unit of enzyme activity.

The method based on Hultmark et al. [24] was improved by using *Micrococcus lysoleikticus* as a substrate to determine lysozyme activity. The substrate was prepared to a certain concentration of suspension (OD570 = 0.3~0.5) with potassium phosphate buffer (0.1 mol/L, pH 6.4). We placed 300 μL of the suspension and 5 μL of serum on the enzyme plate, determine the initial optical density at the 570 nm wavelength (A_0_), then kept it for 30 min in a water bath at 37 °C, and immediately determine the optical density at the 570 nm wavelength (A). The calculation was as follows: lysozyme activity (UL) = (A_0_ − A)/A × 100.

Total RNA was extracted from hepatopancreases and guts of two shrimp randomly selected from each parallel group using Trizol reagent (15596018, Invitrogen, Carlsbad, CA, USA). Analysis of total RNA concentration and quality using a NanoDrop 2000 spectrophotometer (Thermo Fisher Scientific, Waltham, MA, USA). RNA samples with an absorbance (260/280 nm) ranging from 1.8 to 2.1 were used for subsequent analysis. Total RNA (1 μg) from each sample was reverse transcribed into a final volume of 20 μL cDNA with Reverse Transcription Kit (containing dsNase) (Biosharp, Anhui, China). Quantitative real-time PCR (qPCR) was analyzed by ChanQ Universal SYBR qPCR Master Mix (Vazyme Biotech, Nanjing, China), carrying out the specific operational steps according to the manufacturer’s instructions. To verify the stability of gene expression, β-actin was selected as the reference gene [25]. The relative expressions of target genes, including caspase-1 and C-type lectin 3 (CTL3), were analyzed by the 2^−ΔΔCt^ algorithm [26]. The program for the qPCR reaction was 95 °C for 30 s, 40 cycles at 95 °C for 10 s, and 60 °C for 30 s. The sequences of all PCR primers used in this study are given in Table 2.

### 2.5. Gut Microbiota Analysis

Five mixed gut samples were randomly selected from each group to be used to detect gut microbiota. Total genomic DNA from samples was extracted using the CTAB method [27], and agarose gel electrophoresis was used to assess DNA purity and concentration. The V3-V4 region of 16S rRNA genes were amplified by PCR using universal primers 338F (5′ ACTCCTACGGGAGGCAGCA 3′) and 806R (5′ GGACTACHVGGGTWTCTAAT 3′) [28]. The library was built using the TruSeq^®^ DNA PCR-Free Sample Preparation Kit. The library was quality controlled and then sequenced using the NovaSeq6000 (Illumina, San Diego, CA, USA), and bioinformatics analysis of intestinal microbes (microbial composition, diversity, function prediction, interspecific interaction) was completed on the NovoMagic cloud platform (https://magic.novogene.com/customer/main#/tool-micro/28188210446062904461c2e3eebf9034, accessed on 8 March 2021). The sequences obtained in this study are available in the NCBI SRA database with the accession number PRJNA820522.

### 2.6. Gut Microbiota and Biochemical Indexes Association Analysis

Spearman correlation analysis was performed using SPSS software (ver. 26.0; SPSS Inc., Chicago, IL, USA) to show the potential connection between gut microbiota and biochemical pathways and enzymes (such as T-AOC, MDA, GSH-Px, SOD, PO, LZM, CTL3, and caspase-1). This process did not set correlation coefficients and *p*-value thresholds. A Heatmap was used to show the correlation and statistical difference (https://software.broadinstitute.org/morpheus/). Statistical significance is indicated by * *p* < 0.05, ** *p* < 0.01.

### 2.7. Statistical Analysis

Data on growth performance, antioxidant capacity, and immune response analysis were performed using one-way analysis of variance (ANOVA) followed by Duncan’s multiple range test to assess the significance of differences among all experimental treatments. All data are shown as means ± SE (standard error). The student’s *t*-test was used to analyze functional prediction differences between all experimental treatments and control. The value of statistical significance was regarded as *p* < 0.05. All statistical analyses were performed using the SPSS software 26th version (Armonk, NY, USA, IBM SPSS Statistics).

## 3. Results

### 3.1. Growth Performance

**Figure 1:**
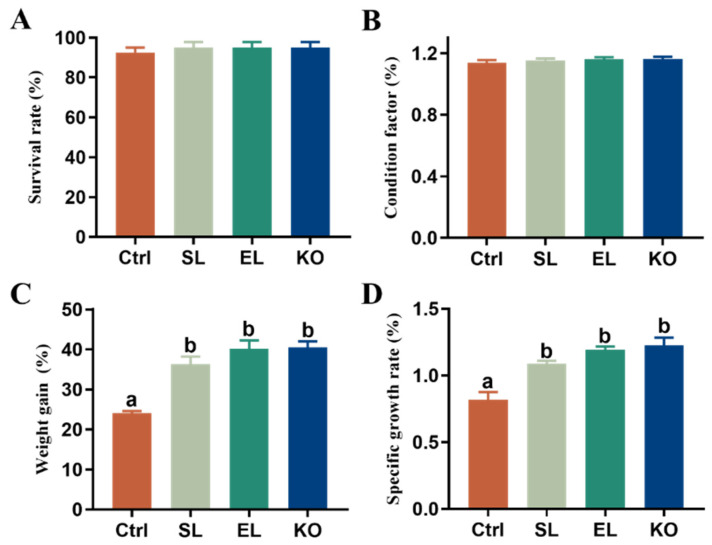
Growth phenotypes of female *L. vannamei* fed different experimental diets. (**A**) Survival rate. (**B**) Condition factor. (**C**) Weight gain. (**D**) Specific growth rate. The values are the mean ± standard errors (*n* = 4). Values with different superscript letters indicate significant differences (*p* < 0.05) among all the treatments.

Relative to the control, dietary phospholipid supplementation significantly increased the weight gain and specific growth rate of female *L. vannamei* broodstock regardless of phospholipid sources, but did not affect survival and condition factor (Figure 1). There were no significant differences in all the growth-related parameters among the SL, EL, and KO groups, although the shrimp fed dietary krill oil showed the highest value for these parameters.

### 3.2. Antioxidant Capacity

**Figure 2:**
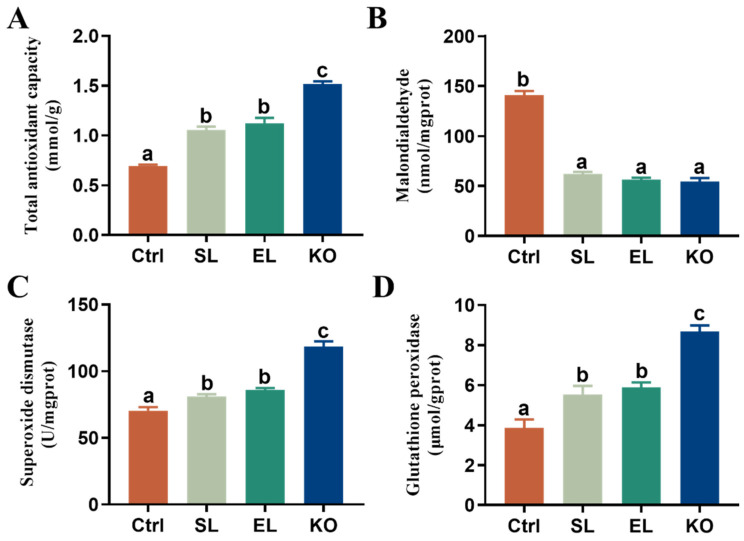
Hepatopancreatic antioxidant ability of female *L. vannamei* fed different experimental diets. (**A**) Total antioxidant capacity (T-AOC). (**B**) Malondialdehyde (MDA). (**C**) Superoxide dismutase (SOD). (**D**) Glutathione peroxidase (GSH-Px). The values are the mean ± standard errors (*n* = 4). Values with superscript different letters indicate significant differences (*p* < 0.05) among all the treatments.

Relative to the control, dietary phospholipid supplementation significantly improved hepatopancreatic T-AOC capacity, activities of SOD and GSH-Px, and decreased the MDA contents in female *L. vannamei* broodstock, regardless of phospholipid sources (Figure 2). Among the three phospholipid-supplemented groups, shrimp fed the KO diet obtained significantly higher T-AOC capacity, activities of SOD, and GSH-Px than shrimp fed SL or EL. However, the MDA contents did not differ among the three groups.

### 3.3. Immune Responses

**Figure 3:**
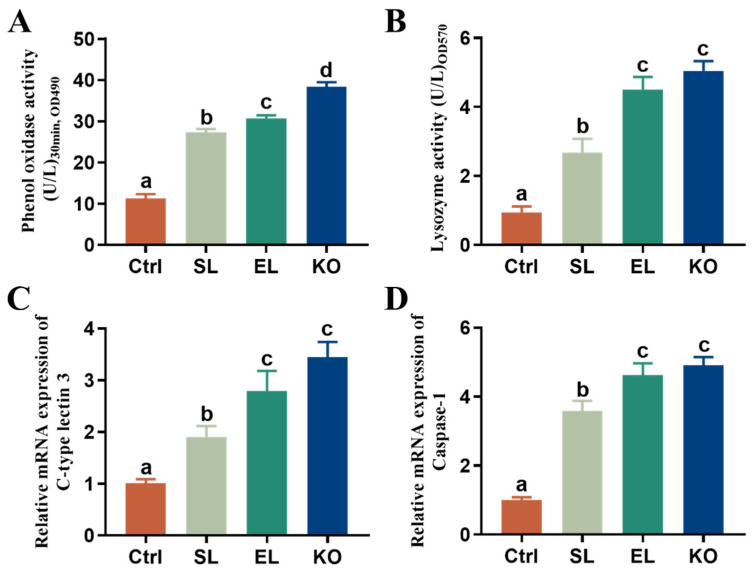
Immune response of female *L. vannamei* fed different experimental diets. (**A**) Phenol oxidase activity in serum. (**B**) Lysozyme activity in serum. (**C**) Relative mRNA expression of C-type lectin 3 in hepatopancreas. (**D**) Relative mRNA expression of Caspase-1 in hepatopancreas. The values are the mean ± standard errors (*n* = 4). Values with superscript different letters indicate significant differences (*p* < 0.05) among all the treatments.

Dietary phospholipid supplementation significantly improved the activities of phenol oxidase and lysozyme and expression of C-type lectin 3 and caspase-1 in female broodstock, regardless of phospholipid sources (Figure 3). Shrimp fed EL or KO showed significantly higher phenol oxidase and lysozyme activities and expression of C-type lectin 3 and caspase-1 than shrimp fed the SL diet. Though shrimp fed the KO diet had higher immunity parameter values than shrimp fed the EL diet, a significant difference was found only for phenol oxidase activity.

### 3.4. Gut Microbiota Analysis

#### 3.4.1. Composition of Gut Microbial Community

**Figure 4:**
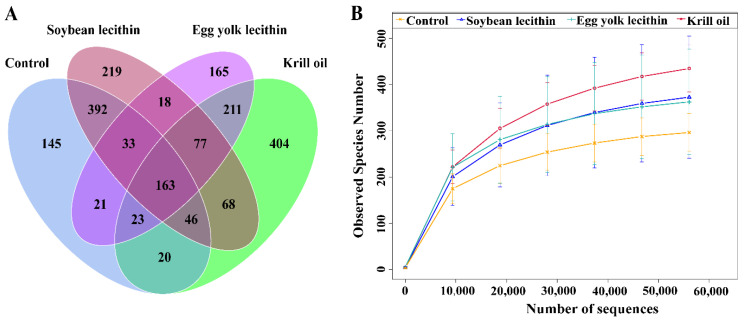
Gut microbial community analysis of female *L. vannamei* fed with different phospholipids. (**A**) Venn diagram indicating the number of unique and shared OTUs. (**B**) Rarefaction curve indirectly reflecting the richness of bacteria in each treatment group.

A total of 2,111,128 high-quality DNA sequences were obtained by gut microbial barcoding, with an average of 105,556 sequences per sample, and 56,215 valid sequences were obtained after quality control, with an average length of 425 bp. The coverage of each sample was higher than 99%. Sequence clusters with a similarity of more than 97% were regarded as belonging to the same Operational Taxonomic Units (OTUs), and 2005 OTUs were obtained. There were 163 OTUs shared among the control group and the respective phospholipid feed groups, accounting for 8.13% of the total OTUs (Figure 4A). In the horizontal direction, the number of sequences was about 6000, indicating that the sequencing was sufficient to cover most taxa in the sample, and as the number of sequences accumulated above 20,000, the rarefication curve tended to plateau, indicating that the more evenly distributed the gut microbial community (Figure 4B).

#### 3.4.2. Diversity of Gut Microbial

**Figure 5:**
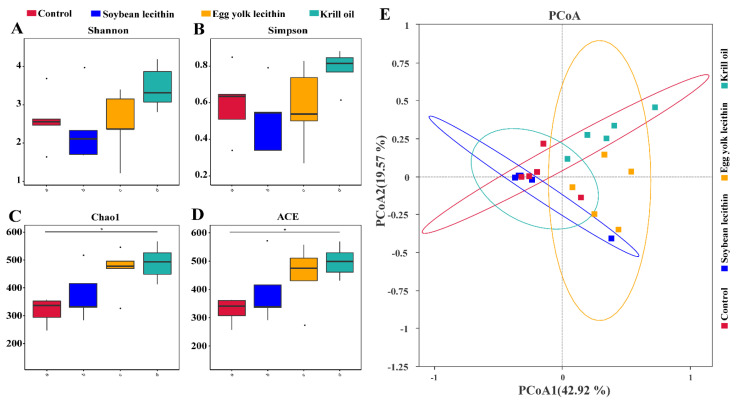
Diversity of gut microbial community of female *L. vannamei* fed with different phospholipids. Alpha diversity indices of bacterial communities on genus level, (**A**) Shannon, (**B**) Simpson, (**C**) Chao1, (**D**) ACE, the * *p* < 0.05 indicate significant differences. (**E**) Beta diversity, the principal coordinates analysis (PCoA) performed to evaluate the overall differences in bacterial community structure based on Bray–Curtis distance.

The α-diversity analysis showed that Shannon and Simpson’s diversity indexes were the highest for the EL and KO groups (Figure 5A,B). The richness indexes Chao 1 and ACE showed that feeding different phospholipids increased gut microbial richness, and that KO supplementation significantly improved gut microbial richness relative to the control (Figure 5C,D). Principal components analysis showed differences in gut microbial community composition among the dietary phospholipid supplementation treatments (Figure 5E).

**Figure 6:**
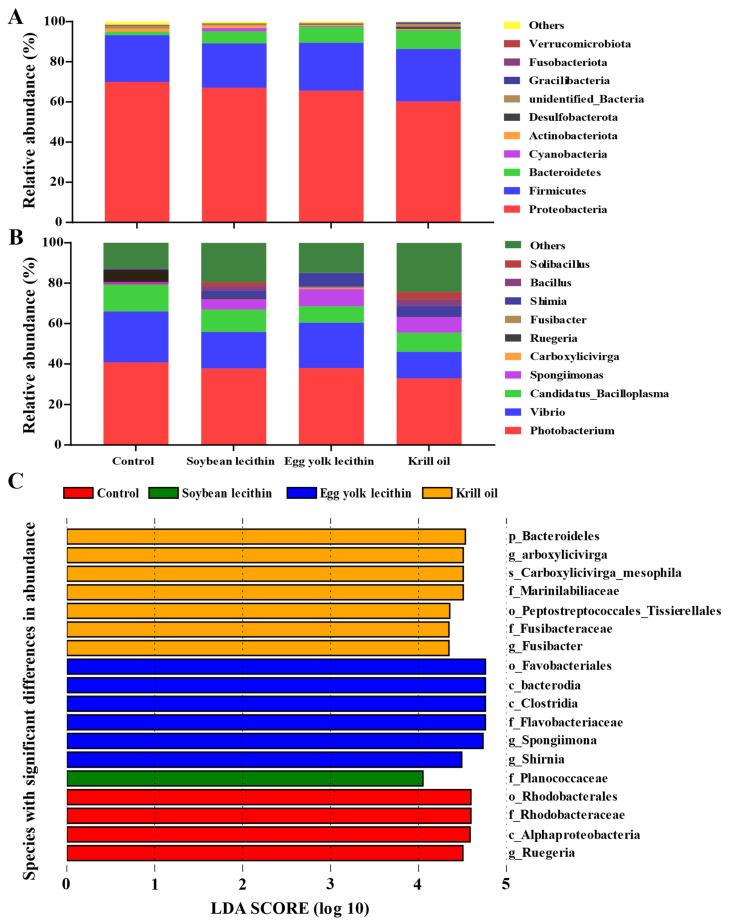
Gut microbial community composition of female *L. vannamei* fed with different phospholipids. Average relative abundances of dominant bacterial phyla (**A**) and genera (**B**) in the intestine. (**C**) Histogram of differentially abundant taxa identified from phylum level to genus level detected by linear discriminant analysis (LDA) effect size analysis (LEfSe; LDA > 3.5).

At the phylum level, the dominant phyla in each group were Proteobacteria, Firmicutes, and Bacteroidetes, and the abundance of the Proteobacteria in the feed phospholipid group differed somewhat among treatments (Figure 6A). Among treatments, the KO group had the lowest abundance of Proteobacteria (60.28%) and the highest abundance of Firmicutes (26.07%) and Bacteroidetes (9.26%). At the genus level, the abundance of *Photobacterium* and *Vibrio* decreased slightly with dietary phospholipid supplementation, and the KO group had the lowest abundance (Figure 6B). Linear discriminant analysis effect size (LEfSe) revealed seven, six, and one biomarkers with significantly higher relative abundance in the KO, EL, and SL groups, respectively, relative to the control (Figure 6C). The phylum Bacteroidetes was significantly more abundant in the gut of shrimp fed the krill oil diet, and the genera *Spongiimonas* and *Shimia* could be regarded as biomarker taxa in the egg yolk lecithin diet. In contrast, the soybean lecithin diet had fewer biomarker taxa, with only the Family Planococcaceae (Figure 6C).

**Figure 7:**
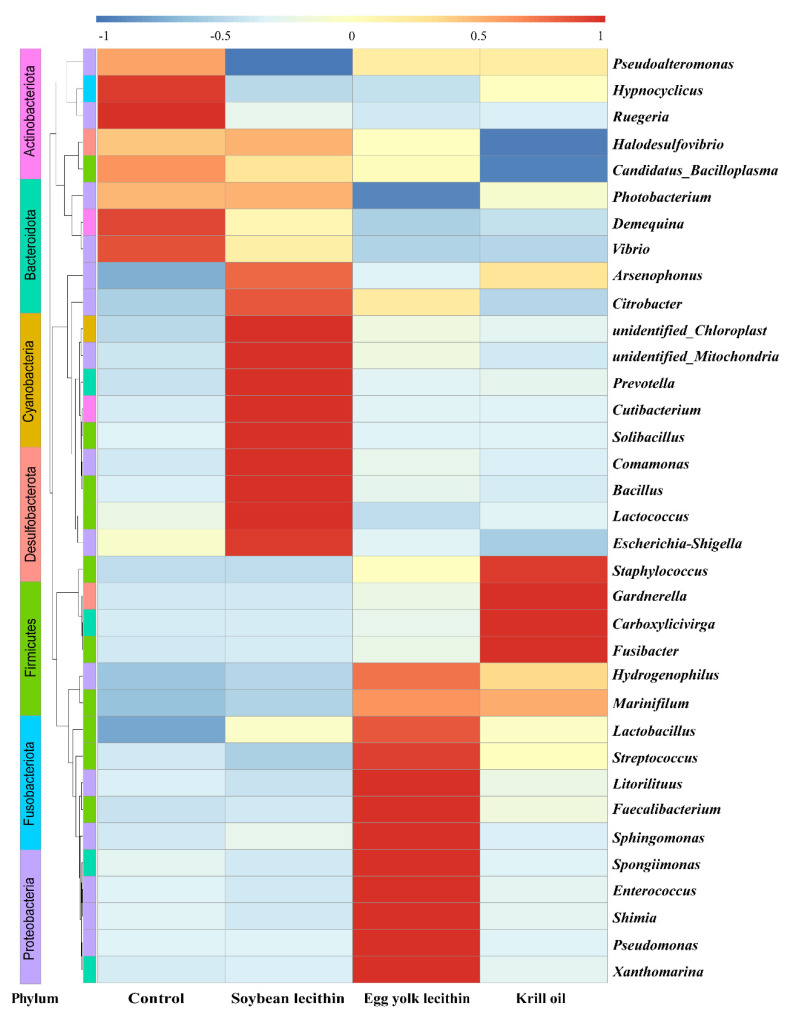
Genus-level composition (top 35) heat map of the gut microbiota of female *L. vannamei* fed different phospholipids. Warmer colors indicate a higher abundance of species.

Comparisons of relative abundance of the top 35 genera detected in the gut of shrimp fed the respective diets were characterized by the construction of a heat map. The heat map showed that the abundance of genera was changed by different dietary phospholipids (Figure 7). In the gut microbial community of the control group, four genera [Fusobacteriota (*Hypnocyclicus*), Proteobacteria (*Ruegeria* and *Vibrio*), and Actinobacteriota (*Demequina*)] were detected. In the SL group, nine genera [Proteobacteria (*Arsenophonus*, *Citrobacter*, *Comamonas*, and *Escherichia-Shigella*), Bacteroidota (*Prevotella*), Actinobacteriota (*Cutibacterium*), and Firmicutes (*Solibacillus*, *Bacillus*, and *Lactococcus*)] were detected. In the EL group, ten genera (Firmicutes (*Lactobacillus*, *Streptococcus*, and *Faecalibacterium*), Proteobacteria (*Litorilituus*, *Sphingomonas*, *Enterococcus*, *Shimia*, and *Pseudomonas*), and Bacteroidota (*Spongiimonas* and *Xanthomarina*)) were detected. In the KO group, four genera (Firmicutes (*Staphylococcus* and *Fusibacter*), Desulfobacterota (*Gardnerella*), and Bacteroidota (*Carboxylicivirga*)) were detected.

#### 3.4.3. Gut Microbiota Functional Prediction

**Figure 8:**
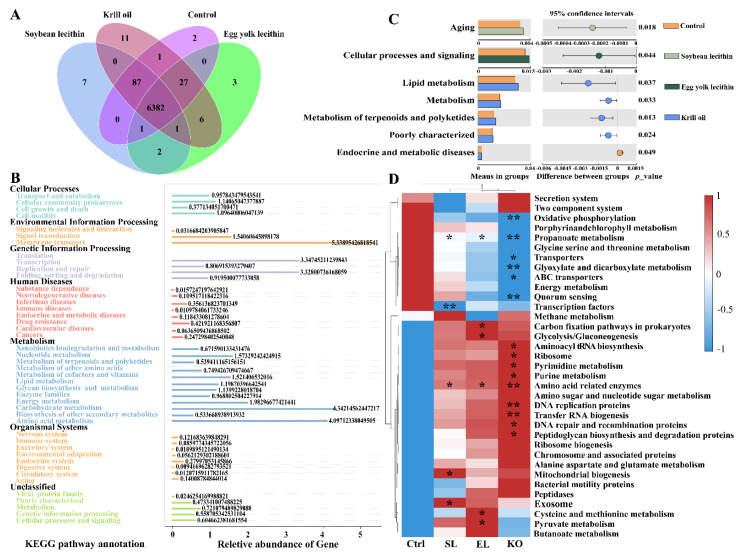
Effect of different phospholipids fed to female *L. vannamei* on gut microbiota function. (**A**) Venn diagram indicates the number of unique and shared OTUs of the gut microbiota. (**B**) Statistical chart of gene prediction results showing the proportion of annotated genes. (**C**) Level-2 functional prediction of gut microbial abundance in different phospholipid groups and control. (**D**) KEGG level-3 clustering heatmap of relative abundance of functions. Asterisks within the different squares indicate the significance of different phospholipid supplementations relative to the control. *p* < 0.05 represents a significant difference, and *T*-test was used for statistical analysis, and * *p* < 0.05, ** *p* < 0.01 indicate statistical significance.

The KO group had the largest number of functional OTUs, followed by the SL and EL groups (Figure 8A). The control and all treatment groups were classified into six categories at KEGG level 1: “Cellular Processes”, “Environmental Information Processing”, “Genetic Information Processing”, “Human Diseases”, “Metabolism”, “Organismal Systems”. Metabolism was the predominant KEGG pathway in all treatment groups (27.3%), including lipid metabolism, energy metabolism, and amino acid metabolism (Figure 8B). At KEGG level 2, relative to the control, there was a significant difference in “Aging” and “Cellular processes and signaling” in the SL and EL groups, respectively (*p* < 0.05) (Figure 8C). Further, relative to the control, the KO group had significant differences in “Lipid metabolism”, “Metabolism”, and “Metabolism of terpenoids and polyketides” (*p* < 0.05) (Figure 8C). At KEGG level 3, relative to the control, “Amino acid related enzymes” were significantly increased in phospholipid supplementation groups (*p* < 0.05) (Figure 8D).

#### 3.4.4. Gut Microbiota Network

**Figure 9:**
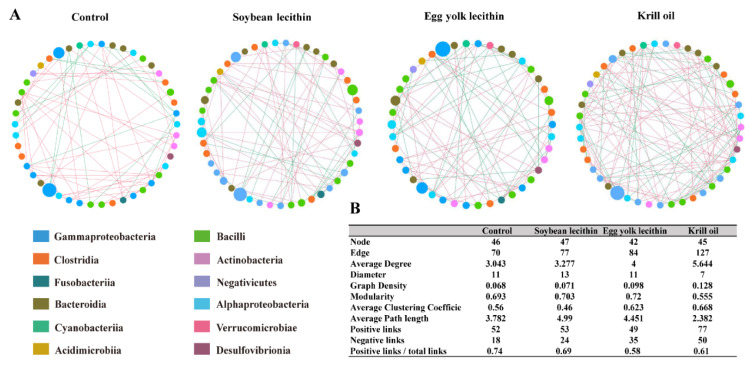
Ecological interaction network analysis of gut microbial community. (**A**) Interspecies interaction network of bacteria communities for female *L. vannamei* fed with different phospholipids. Each node represents a genus. Node colors indicate genus affiliated with different classes. A green edge indicates negative correlations between two individual nodes, whereas a red edge indicates positive correlations. (**B**) Topological properties of gut microbial community networks.

According to the analysis of the interspecies interaction network of gut microbial communities (Figure 9A,B), the control group was composed of 46 nodes and 70 edges. The network diagram for the SL group was composed of 47 nodes and 77 edges. The network diagram of the EL group was composed of 42 nodes and 84 edges. The network diagram for the KO group was composed of 45 nodes and 127 edges. Relative to the control, the number of “edges” in the network diagram for each phospholipid group increased, and the number for the KO group was the largest. In addition, the average degree and average clustering coefficient for each phospholipid group were higher than those of the control group. The analysis showed that the gut microbial community in the KO group had the most complex interspecific relationships and the greatest positive interactions.

### 3.5. Gut Microbiota and Biochemical Indexes Association Analysis

**Figure 10:**
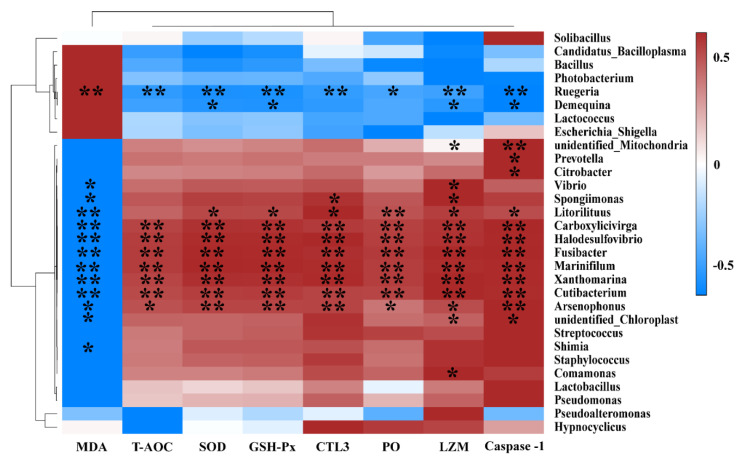
Correlation analysis between genera of gut microbiota and biochemical indexes. Red squares indicate positive correlations, whereas blue squares indicate negative correlations. Asterisks within the different squares indicate significance, * *p* < 0.05, ** *p* < 0.01.

Results of the microbiota and biochemical indexes association analysis at the genus level are shown in Figure 10. *Carboxylicivirga*, *Halodesulfovibrio*, *Fusibacter*, *Marinifilum*, *Xanthomarina*, and *Cutibacterium* were positively correlated with T-AOC, SOD, GSH-Px, CTL3, PO, LZM, and Caspase-1, respectively, and negatively correlated with MDA (*p* < 0.01). *Ruegeria* was positively correlated with MDA and negatively correlated with T-AOC, SOD, GSH-Px, CTL3, LZM, and Caspase-1 (*p* < 0.01). By contrast, *Arsenophonus* and *Litorilituus* were positively correlated with SOD, GSH-Px, CTL3, PO, LZM, and Caspase-1, but negatively correlated with MDA (*p* < 0.05). *Spongiimonas*, *Vibrio*, and *Comamonas* were positively correlated with LZM, and *Prevotella* and *Citrobacter* were positively correlated with Caspase-1 (*p* < 0.05). In contrast, *Demequina* was negatively correlated with GSH-Px, SOD, LZM, and Caspase-1, and *Vibrio*, *Spongiimonas*, and *Shimia* were negatively correlated with MDA (*p* < 0.05).

## 4. Discussion

Maintaining the health of *L. vannamei* broodstock is particularly important for the industry’s sustainable development. Studies have shown that dietary phospholipid supplementation can significantly improve the growth performance of crustaceans and fishes [29,30]. Similar results were found in this study. Adding phospholipids to diets significantly increased shrimp weight gain and specific growth rates. Further, the growth performance of shrimp fed with krill oil was better than that fed soybean lecithin and egg yolk lecithin. The same results were obtained in the study of *E. sinensis* [31]. The possible reason is that krill oil has a higher n-3 HUFA content. Studies have shown that the replacement of 50–100% soybean oil with dietary black soldier fly oil rich in n-3 HUFA has a positive effect on the growth performance and health of juvenile mirror carp (*Cyprinus carpio* var. *specularis*) [32], and the first stages of juvenile *L. vannamei* fed the diet containing 0.86 % n-3 HUFA had highest weight gain and specific growth rate [33]. Krill oil is a good source of n-3 phospholipids with high bioavailability [34], which explains how feeding krill oil can achieve the highest growth performance. The effects of phospholipids on aquatic animals vary with the phospholipids’ dosages and forms [31,35], affecting the immune and antioxidant systems that are the two primary physiological mechanisms protecting the health of aquatic animals [36].

Dietary phospholipids can enhance the animal’s ability to resist environmental stress and induce an antioxidant response to protect organs from oxidative damage [37,38]. The antioxidation mechanism of phospholipids can be a function of enzymatic oxidation or non-enzymatic oxidation, which is initiated by reactive oxygen species and mediated by the free radical chain reaction [39]. This study showed that the dietary phospholipids significantly increased shrimp’s total antioxidant capacity (T-AOC). T-AOC is an important indicator of the antioxidant system for scavenging excessive reactive oxygen species (ROS) [40]. ROS is mainly produced in the hepatopancreas of crustaceans [41,42]. Studies have shown that the n-3 polyunsaturated fatty acids (n-3 PUFAs) in the organism are extremely easy to oxidize and induce reactive oxygen species production [42,43]. Many antioxidant enzymes are produced in an organism to reduce the detrimental effect of reactive oxygen species, including superoxide dismutase (SOD) and glutathione peroxidase (GSH-Px) [38,44]. In addition, one of the important biomarkers of oxidative stress injury has been considered to be malondialdehyde (MDA), a product of lipid peroxidation [42,43]. Dietary phospholipids can significantly increase the specific activity of superoxide dismutase (SOD) and glutathione peroxidase (GSH-Px) and reduce the content of malondialdehyde (MDA) [37,45,46]. This study confirms the view that dietary phospholipids can significantly increase the activities of SOD and GSH-Px in the hepatopancreas. Female shrimp with gonadal development accumulate much fat in the hepatopancreas, and the activities of SOD and GSH-Px are significantly increased, thereby reducing the content of MDA. As a result, dietary phospholipids can improve the oxidative pressure of excessive fat accumulation in the hepatopancreas. Our previous research has shown that differentially expressed genes could enrich glutathione metabolic pathways after feeding different phospholipid diets, confirming the hypothesis that dietary phospholipids can improve antioxidant capacity [21]. Furthermore, krill oil showed a greater antioxidant effect than soy lecithin and egg yolk lecithin. It is believed that krill oil contains an effective antioxidant, astaxanthin [47].

Due to a lack of adaptive immunity, crustaceans can only rely on innate immunity to remove pathogenic microorganisms [48], and it is the first line of defense against pathogenic infections [49]. The innate immunity of crustaceans includes cellular immunity and humoral immunity [50]. Apoptosis plays a crucial role in impeding viral propagation by eliminating infected cells [51]. Caspase-induced apoptosis can inhibit white spot syndrome virus (WSSV) infection by innate immunity in shrimp [52]. In this study, dietary phospholipids significantly up-regulated the relative mRNA expression of caspase-1 in hepatopancreas. We suggest that the diet supplemented with phospholipids can increase the relative mRNA expression of caspase-1 and induce apoptosis as appropriate to reduce the risk of WSSV infection in shrimp. Phenoloxidase (PO) [53] and lysozyme (LZM) [54] play essential roles in the nonspecific immune system of crustaceans and can eliminate foreign pathogenic microorganisms by damaging acetaminoglycan in the cell walls of Gram-positive bacteria. Other humoral immune factors playing important roles in the immune response of crustaceans include lectins [55], which can bind to carbohydrates on the surface of pathogens and elicit antimicrobial responses in shrimp. This study shows that dietary phospholipids can enhance the activities of PO and LZM and up-regulate the expression of CTL3. Therefore, based on the study results, we suggest that the intake of phospholipid in shrimp feed can enhance the immune system to resist the invasion of pathogens.

The gut is a suitable environment for the colonization and proliferation of symbiotic microorganisms in aquatic animals [56]. The intestine is the main organ for digestion and absorption of nutrients and the largest “immune organ” of the organism [57]. The gut microbiota promotes gut health and ensures the continuous normal physiological function of the gut by constructing the first barrier against pathogens [58]. Proteobacteria, Bacteroidetes, Actinobacteria, and Firmicutes are dominant in the shrimp gut at various stages of development [59]. Similarly, in this study, the dominant bacteria in each group were Proteobacteria, Firmicutes, and Bacteroidetes, and dietary phospholipids decreased the abundance of Proteobacteria in the gut. Proteobacteria include *Rickettsiaceae* which is a pathogenic bacterium that endangers the health of shrimp [60]. Firmicutes and Bacteroidetes can participate in maintaining gut immune homeostasis and improve the health of the animals [61]. These results suggest that dietary phospholipid can benefit intestinal immune homeostasis in shrimp. By contrast, dietary krill oil induced a reduction in the abundance of Proteobacteria in this study.

Krill oil is a significant source of gut-derived endotoxin lipopolysaccharide (LPS) [62] to trigger inflammation by innate immune responses in the liver [63]. Our results show that dietary krill oil could reduce hepatopancreatic injury caused by Proteobacteria. At the genus level, *Fusibacter* is associated with iron metabolism [64], and the metabolites participate in the antioxidant and immune responses in the form of ferritin [65,66]. The present study shows that the addition of KO to the feed could increase the richness of *Fusibacter* compared to the addition of SL and EL. *Fusibacter* was positively associated with antioxidant (T-AOC, SOD, GSH-Px) and immune response (CTL3, PO, LZM, Caspase-1). The level of gene expression ferritin can be upregulated in yellowhead virus infection of *Penaeus monodon* and acute viral attack of *Chlamys farreri* [67,68]. It is hypothesized that dietary phospholipids increase the richness of *Fusibacter*, possibly by participating in iron metabolism and thus increasing the antioxidant and immune responses of female shrimp. The abundance of the microbiome is an important environmental factor affecting energy uptake and storage in the gut [69]. Gut microbes form complex ecological networks through cooperation, competition, predation, and other interactions [70]. Furthermore, excessive n-6 HUFA may interfere with gut microbial homeostasis [58]. In this study, the n-6 HUFA in the KO diet was significantly lower than those in the SL and EL groups, indicating that KO can strongly regulate the homeostasis of intestinal microbiomes. Adding KO to the feed can enhance the function of gut microbial genes, enrich the “lipid metabolism” and “energy metabolism” pathways and enhance the inter-species interactions between microorganisms. These results indicate that feed with KO can promote the stability of the gut microbial community and maintain the health of shrimp.

## 5. Conclusions

Adding phospholipids to the diet can improve the antioxidant capacity of *L. vannamei* broodstock and improve natural immunity to resist environmental pathogenic bacteria. Especially with krill oil as a source of phospholipids, the activities of SOD and GSH-Px in the hepatopancreas were increased, and the total antioxidant capacity of female shrimp was enhanced. In addition, the diet supplemented with krill oil could enhance the interaction of gut microbiota of female shrimp and increase the abundance of *Fusibacter* within Firmicutes. More importantly, *Fusibacter* may be involved in iron metabolism to improve the antioxidant capacity of female shrimp. Furthermore, krill oil can help establish the gut immune barrier to enhance the immune response of female shrimp, reduce the risk of pathogenic bacteria infection and maintain the healthy growth of female shrimp.

## Figures and Tables

**Table 1:** Formulation (g/kg dry basis), proximate composition (%), and statistical analysis of differences for PUFAs contents of the experimental diets fed to female *L. vannamei*.

Ingredients	Experimental Diets			
	Ctrl	SL	EL	KO
Fish meal	200	200	200	200
Casein	320	320	320	320
Gelatin	80	80	80	80
Corn starch	150	150	150	150
Fish oil	10	10	10	10
Soybean lecithin	0	40	0	0
Egg yolk lecithin	0	0	40	0
Krill oil	0	0	0	40
Cholesterol	5	5	5	5
Palm oil	80	40	40	40
Butylated hydroxytoluene	1	1	1	1
Anhydrous calcium carbonate	4	4	4	4
Calcium lactate pentahydrate	4	4	4	4
Choline chloride	5	5	5	5
Inositol	0.25	0.25	0.25	0.25
Betaine	20	20	20	20
Vitamin premix ^1^	10	10	10	10
Mineral premix ^2^	20	20	20	20
Carboxymethyl cellulose	20	20	20	20
Cellulose	70.75	70.75	70.75	70.75
Total	1000	1000	1000	1000
**Analyzed proximate composition (%)**				
Moisture	7.51	7.43	7.78	7.42
Crude protein	52.33	52.15	52.50	52.47
Crude lipid	14.28	14.40	14.05	14.22
Ash	8.31	8.30	8.28	8.31
n-3 PUFAs	495.31 ^a^	694.01 ^c^	568.93 ^b^	2335.27 ^d^
n-6 PUFAs	335.62 ^a^	1577.41 ^d^	577.74 ^c^	399.4 ^b^

The ^1^ vitamin premix and ^2^ mineral premix are formulated with reference to the formulation suitable for female *L. vannamei* in our laboratory [21]. Values are means ± SE (*n* = 3) and values within a row with different superscript letters (a, b, c, d) are significantly different (*p* < 0.05).

**Table 2:** Primer-pair sequences and product size of the amplicons used for quantitative real-time PCR (qPCR).

Gene	Primer Sequence	Tm (°C)	GC%	GenBank NO
β-actin	F: GCAGTCCAACCCGAGAGGAAG	61.49	62.00	XM_027364954
	R: GTGCATCGTCACCAGCGAA	57.09	58.00	
caspase-1	F: CGGGTAGGAAGCCCACATATCAA	59.78	52.00	XM_027356206
	R: ACGGCGAAGTCAAAGCCAGAA	57.59	52.00	
CTL3	F: ATGTTCTTCGTGCTCCTGCTGT	57.80	50.00	XM_027356524
	R: GCAGTGGTCGTAAATGTTGTG	55.63	48.00	

## Data Availability

The data provided in this study have been uploaded to the NCBI database. The accession number is PRJNA820522, and the link is https://www.ncbi.nlm.nih.gov/sra/PRJNA820522 (accessed on 29 March 2022).
